# Supplementary figures and images for: Prediction of heterosis in the recent rapeseed (Brassica napus) polyploid by pairing parental nucleotide sequences
Source: PLoS Genet. 2021 Nov 4;17(11):e1009879. doi: 10.1371/journal.pgen.1009879 (PMC8608326; doi:10.1371/journal.pgen.1009879)

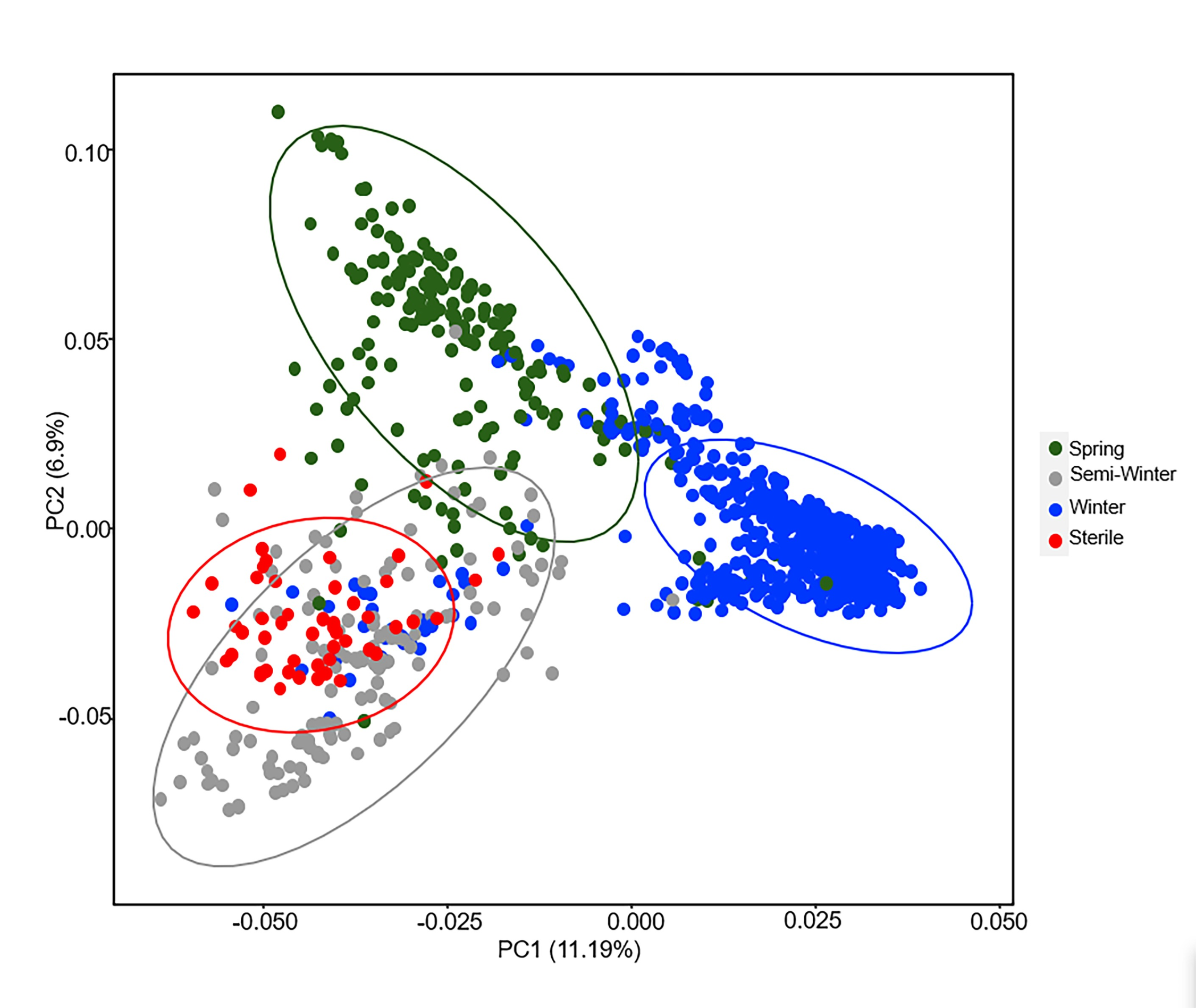

Supplement: S1 Fig — PC1 accounts for 11.19% of the total variation in the winter-type accessions compared to the other accessions, whereas PC2 accounts for 6.90% of the total variation between the semi-winter type and the spring type accession. Green dots represent spring ecotype, blue dots represent winter ecotype, grey dots represent semi-spring ecotype, and red dots represent sterile lines that were used as female parents in the study. (TIF) [file pgen.1009879.s001.tif]

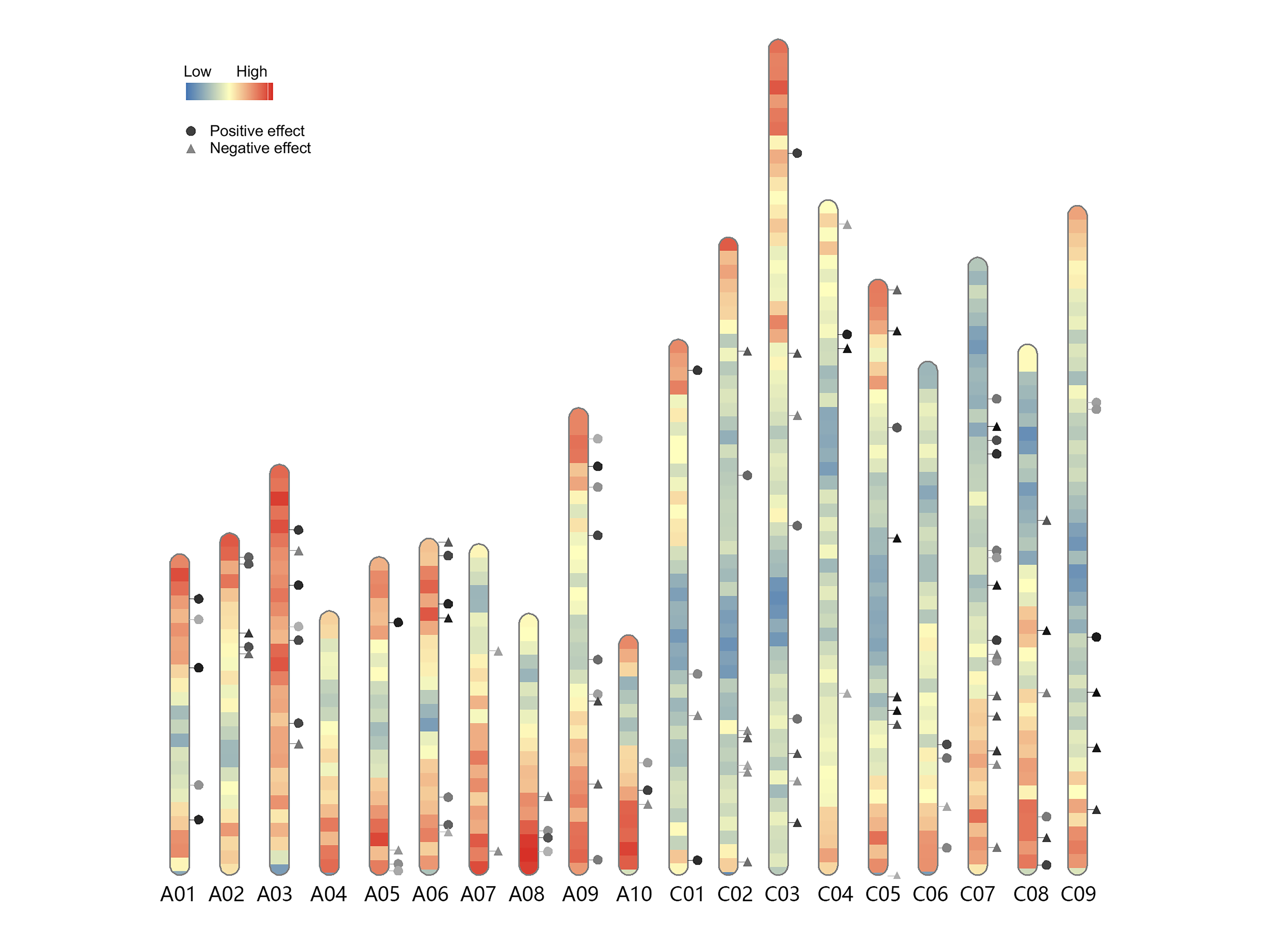

Supplement: S2 Fig — The circles represent the h-QTLs that positively contributed to the TSW-HPH, and the triangles represent h-QTLs that were negatively correlated to TSW-HPH. The darker the colors of circles and triangles, the greater the effects of the h-QTLs, either positive or negative. The colors on the chromosomes indicate the density of genes. The darker the blue, the lower the gene density, the darker the red, the higher the gene density. A and C stand for the two sub-genomes of Brassica napus. A limited number of h-QTLs on randomly piled contigs, whose positions on certain chromosomes were unknown, are not shown on the map. A positive effect indicated with a circle on maps means the smaller the PGSI, the great the heterosis, whereas, a negative effect tagged with a triangle means the bigger the PGSI, the greater the heterosis. (TIF) [file pgen.1009879.s002.tif]

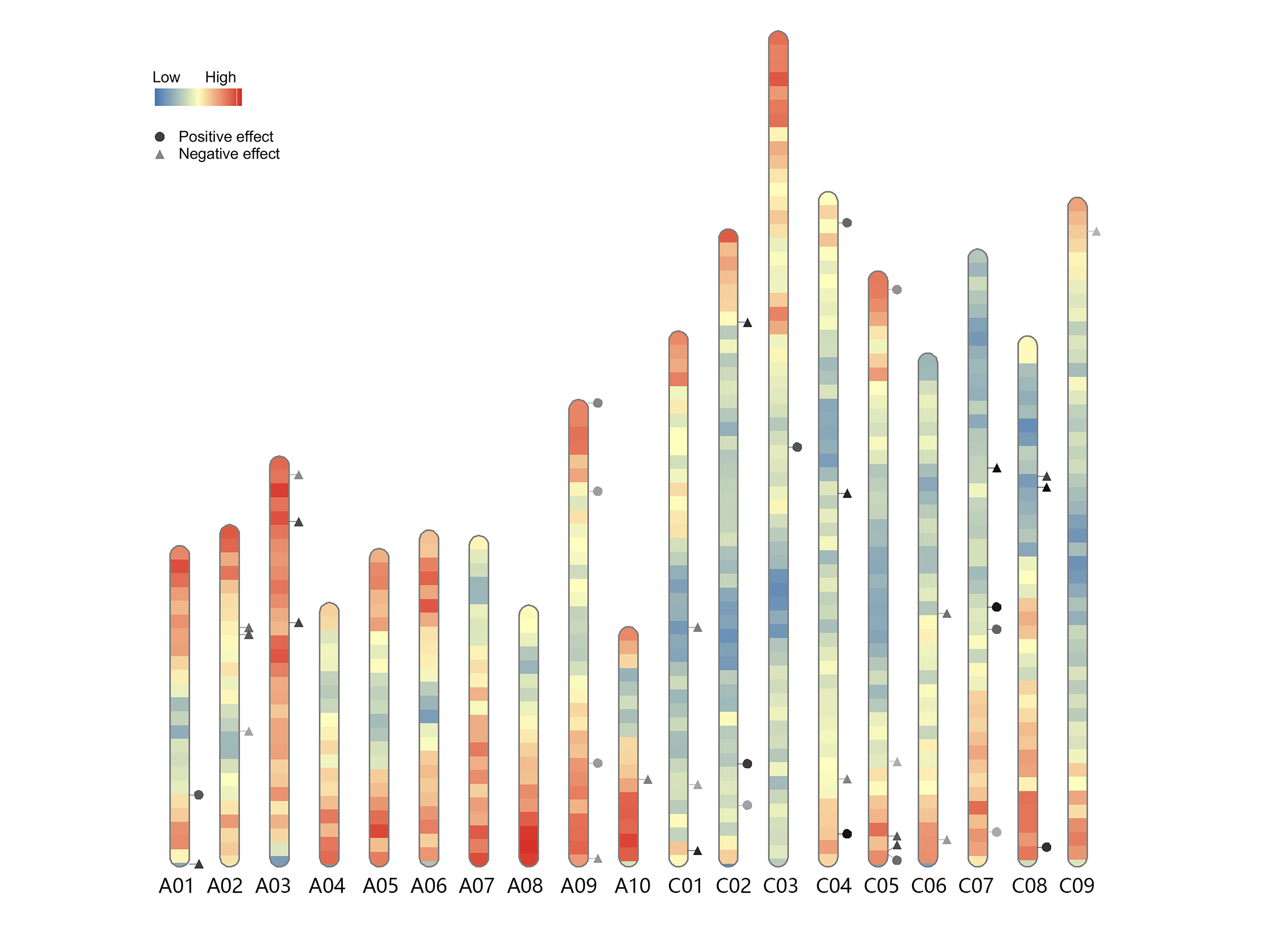

Supplement: S3 Fig — The circles represent the h-QTLs that positively contributed to the NSS-HPH, and the triangles represent h-QTLs that were negatively correlated to NSS-HPH. The darker the colors of circles and triangles, the greater the effects of the h-QTLs, either positive or negative. The colors on the chromosomes indicate the density of genes. The darker the blue, the lower the gene density, the darker the red, the higher the gene density. A and C stand for the two sub-genomes of Brassica napus. A limited number of h-QTLs on randomly piled contigs, whose positions on certain chromosomes were unknown, are not shown on the map. A positive effect indicated with a circle on maps means the smaller the PGSI, the great the heterosis, whereas, a negative effect tagged with a triangle means the bigger the PGSI, the greater the heterosis. (TIF) [file pgen.1009879.s003.tif]

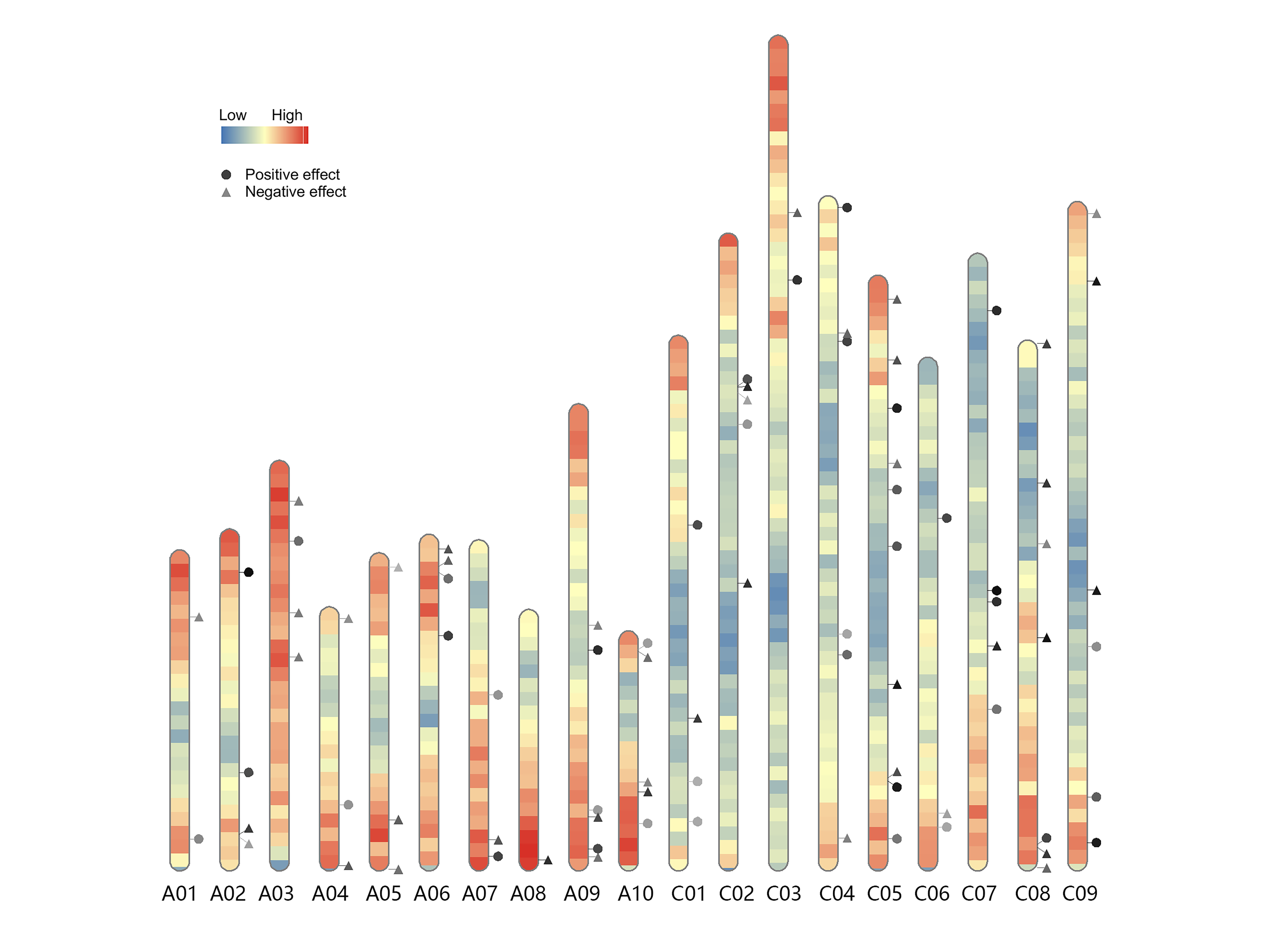

Supplement: S4 Fig — The circles represent the h-QTLs that positively contributed to the NSP-HPH, and the triangles represent h-QTLs that were negatively correlated to NSP-HPH. The darker the colors of circles and triangles, the greater the effects of the h-QTLs, either positive or negative. The colors on the chromosomes indicate the density of genes. The darker the blue, the lower the gene density, the darker the red, the higher the gene density. A and C stand for the two sub-genomes of Brassica napus. A limited number of h-QTLs on randomly piled contigs, whose positions on certain chromosomes were unknown, are not shown on the map. A positive effect indicated with a circle on maps means the smaller the PGSI, the great the heterosis, whereas, a negative effect tagged with a triangle means the bigger the PGSI, the greater the heterosis. (TIF) [file pgen.1009879.s004.tif]

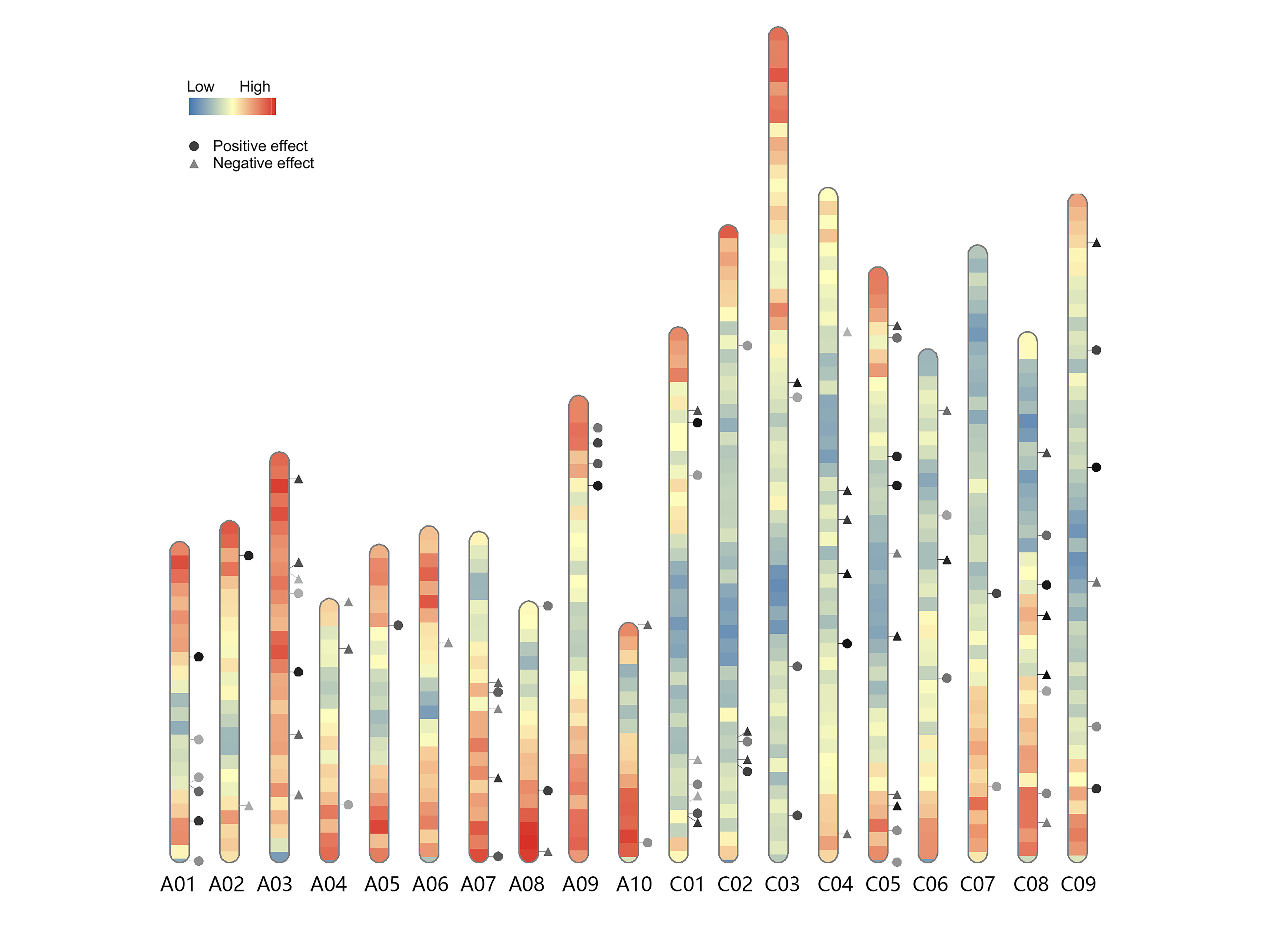

Supplement: S5 Fig — The circles represent the h-QTLs that positively contributed to the NBP-HPH, and the triangles represent h-QTLs that were negatively correlated to NBP-HPH. The darker the colors of circles and triangles, the greater the effects of the h-QTLs, either positive or negative. The colors on the chromosomes indicate the density of genes. The darker the blue, the lower the gene density, the darker the red, the higher the gene density. A and C stand for the two sub-genomes of Brassica napus. A limited number of h-QTLs on randomly piled contigs, whose positions on certain chromosomes were unknown, are not shown on the map. A positive effect indicated with a circle on maps means the smaller the PGSI, the great the heterosis, whereas, a negative effect tagged with a triangle means the bigger the PGSI, the greater the heterosis. (TIF) [file pgen.1009879.s005.tif]

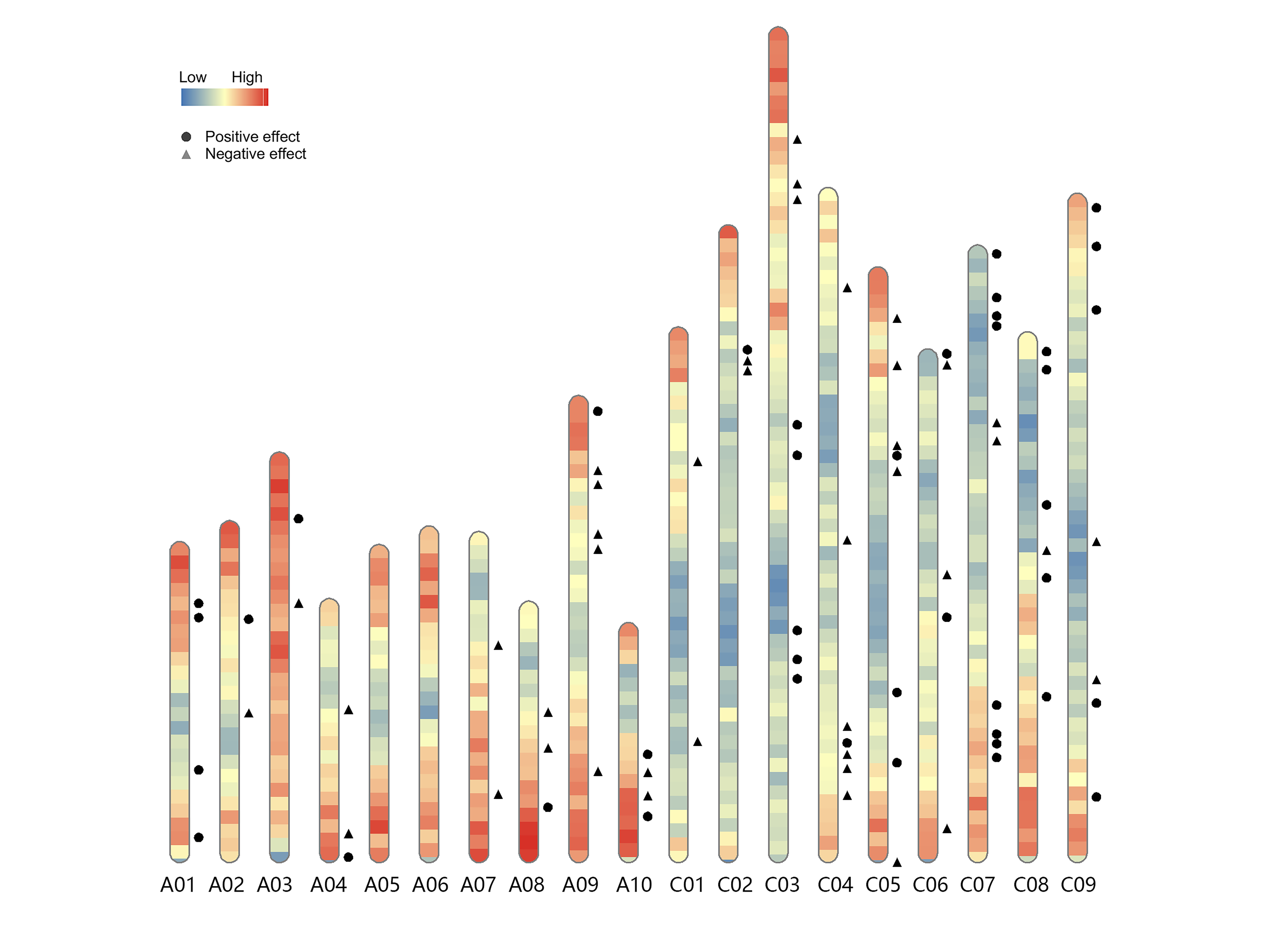

Supplement: S6 Fig — The circles represent the h-QTLs that positively contributed to the PH-HPH, and the triangles represent h-QTLs that were negatively correlated to PH-HPH. The darker the colors of circles and triangles, the greater the effects of the h-QTLs, either positive or negative. The colors on the chromosomes indicate the density of genes. The darker the blue, the lower the gene density, the darker the red, the higher the gene density. A and C stand for the two sub-genomes of Brassica napus. A limited number of h-QTLs on randomly piled contigs, whose positions on certain chromosomes were unknown, are not shown on the map. A positive effect indicated with a circle on maps means the smaller the PGSI, the great the heterosis, whereas, a negative effect tagged with a triangle means the bigger the PGSI, the greater the heterosis. (TIF) [file pgen.1009879.s006.tif]

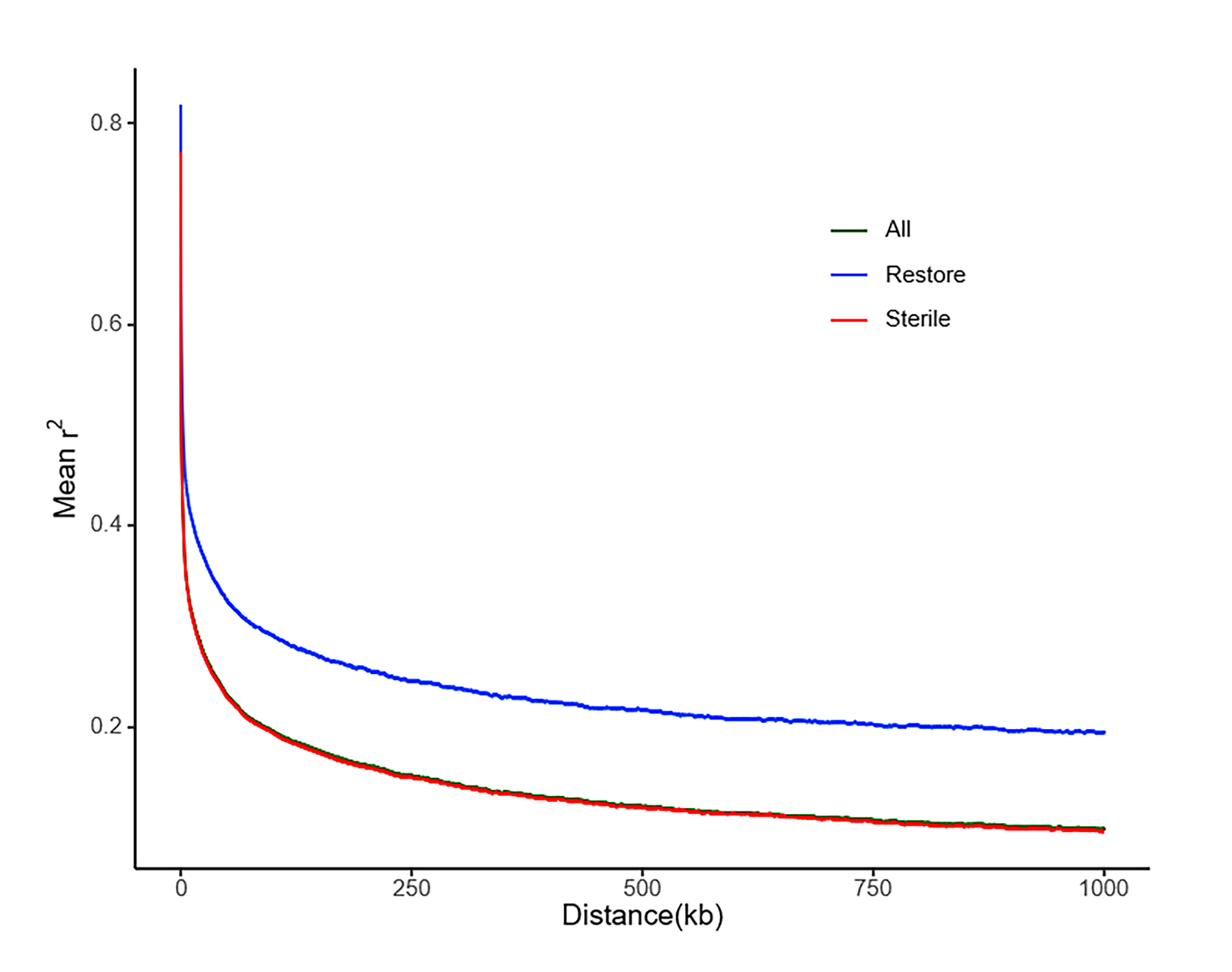

Supplement: S7 Fig — The green, red, and blue curves display the rate of LD decay over distance(Kb) in all sixty parental lines, sterile lines, and restore lines, respectively. (TIF) [file pgen.1009879.s007.tif]
