# Supplementary material for: Prediction of heterosis in the recent rapeseed (Brassica napus) polyploid by pairing parental nucleotide sequences
Source: PLoS Genet. 2021 Nov 4;17(11):e1009879. doi: 10.1371/journal.pgen.1009879 (PMC8608326; doi:10.1371/journal.pgen.1009879)
Supplement: S1 Data — (DOCX) [file pgen.1009879.s030.docx]

**S1 Data.** **Code to build the model and obtain the predictability of the model.**

pacman::p_load(

# basic syntax

dplyr, magrittr,

# load data

readr, readxl,

# data.table for big data

data.table,

# ggplot2 for aesthetic plots

ggplot2, export,

# regression and output utilities

glmnet, lfe, stargazer)

# define root path to be directory of source file

# ! caution: complications may arise when the file is sourced

# ! and/or when knitr is used

# load data

df_name <- read_csv("Names.csv") %>% as.data.table()

df <- read_csv(file = "Train.csv") %>% as.data.table()

g_attributes <- names(df)[(ncol(df) - 13):(ncol(df) - 2)]

g_labels <- names(df)[(ncol(df) - 13):(ncol(df) - 2)]

times <- seq(from=101,to=200)

for (t in 1:100){

time = times[t]

set.seed(time)

# LASSO ----

# flags

lambda_plot <- T

coef_plot <- T

pred_plot <- T

min_regressor <- 1

# loop

# loop through outcomes

g_attribute <- g_attributes[i]

g_label <- g_labels[i]

# drop observations with missing labels

df_tmp <- df[!is.na(df[[g_attribute]])]

# extract feature matrix and outcome vector

feature <- df_tmp[, 1:(ncol(df) - 14)] %>% as.matrix()

outcome <- df_tmp[[g_attribute]]

# fit model

m <- cv.glmnet(x=feature, y=outcome, nfolds=10)

# output to be plotted

tmp <- data.frame(non_zero=m$nzero, cv_mse=m$cvm)

# use the default lambda.min except when

# the number of regressors selected are too small

# (so the model is only predicting mean)

lambda <- m$lambda.min

# number of regressors at lambda.min

j <- (coef(m, s="lambda.min") != 0) %>% sum()

# impose restriction min_regressor

if (j < min_regressor) {

j <- min_regressor

# biggest lambda that yields the number of regressors = min_regressor

lambda <- m$lambda[which(m$nzero <= min_regressor) %>% max()]

}

# extract coefficients

tmp <- coef(m, s=lambda)

tmp <- data.frame(feature=dimnames(tmp)[[1]], coef=matrix(tmp))

# drop constant

tmp <- tmp[2:nrow(tmp), ]

# drop zero coefficients

tmp$coef[tmp$coef == 0] <- NA

# parse feature names

tmp <- merge(tmp, df_name, by="feature")

# re-estimate with linear regression

tmp_names <- tmp[!is.na(tmp$coef), "feature"] %>% as.character()

m <- lm(

as.formula(g_attribute %>% paste0(" ~ ") %>%

paste0(paste(tmp_names, collapse=" + "))), df_tmp)

tmp_merge <- data.frame(beta=m$coefficients,

feature=names(m$coefficients),

row.names=NULL)

names(tmp_merge) <- c("beta", "feature")

tmp <- merge(tmp, tmp_merge, by="feature", all.x=T)

tmp$newlabel <- ifelse(is.na(tmp$beta),tmp$beta,tmp$start_loc / 1e+6)

write.csv(tmp, file = time %>% paste0(g_attribute) %>% paste0(".csv"),row.names = FALSE)

# plot predicting scatter plot

tmp <- data.frame(true=outcome, pred=predict(m))

names(tmp) <- c("true", "pred")

p <- ggscatter(tmp,

x = "true",

y = "pred",

add = "reg.line",

add.params = list(color = "black",fill = "lightgray"),

conf.int = TRUE,

cor.coef = TRUE,

cor.method = "pearson"

)

print(p)

ggsave(file = time %>% paste0("_") %>% paste0("lasso_r2_") %>% paste0(g_attribute) %>% paste0("_") %>% paste0(".pptx"), width=6, height=4)

}
