# Supplementary material for: Prediction of heterosis in the recent rapeseed (Brassica napus) polyploid by pairing parental nucleotide sequences
Source: PLoS Genet. 2021 Nov 4;17(11):e1009879. doi: 10.1371/journal.pgen.1009879 (PMC8608326; doi:10.1371/journal.pgen.1009879)
Supplement: Attachment — Submitted filename: Responses to reviewers.docx [file pgen.1009879.s031.docx]

**Responses to reviewers**

**Reviewer 1**

**>>>General comment**

Heterosis is very important in agricultural productions. This work addressed yield heterosis in rapeseed by crossing 50 CMS accessions and 8 restorers. The population was then used for genomic selection. The work may provide useful resources for future heterosis sutdies. Below are suggestions for the work:

**R:** Thank you so much for the positive comment in general about the importance of our work.

**>>>>Q1**

1. Actually, the sample size (n=400) is not enough for yield traits. Any discussions?

**A1:** We added some discussions about the sample size of our experiment (Lines 447-451). We would think that the field experiment with 400 hybrids in three replicates was not a small scale for rapeseed. Moreover, the CMS pool consisting of maternal lines was genetically diverse, the population that contained 400 hybrids demonstrated a wide range of heterosis in all the six agronomic traits investigated.

**>>>Q2**

2. GO analyses should be removed. Such analyses applied in this paper are misleading! In one interval, there are tens of genes, and only one of them are the causal one. Pooling tens of "false" genes and a correct one for GO must lead to wrong conclusions.

**A2:** We removed GO analyses in the main text according to the suggestion. However, we remained the potentially responsible genes in a supplementary table for interested readers. The average size for LD distance in rapeseed was less than 100Kb. An *h*-QTL of 100 Kb in size might span across 2 linked groups on average. There must be a few genes that were responsible for a kind of heterosis despite the ‘false’-gene majority. We discussed this point in the new version (Lines 338-344).

**>>>Q3**

3. No independent validation crosses for the modelling. The authors should generate tens of crosses (not from 50 CMS accessions and 8 restorers) and tested the accuracy of their GS model.

**A3**: We generated 100 crosses (50 x 2) that were independent of the cross-validation for the validation（Lines 516-518）. Since the breeding for an *ogu*-restorer was extremely time-costing and laborious, we only had two independent restorers for the crosses. The result showed that the model could successfully indicate a superior restorer for the traits such as plant height, thousand-seed weight, and the number of seeds per silique. However, the model could not tell a superior restorer but only superior parental combinations for the rest of the traits such as grain yield (GY), number of branches per plant (NBP), and number of siliques per plant (NSP) (Lines 280-290).

**>>>Q4**

4. The genotypic and phenotypic data should be publically available for authors.

**A4**: All raw reads of the rapeseed accessions generated in this study have been deposited in the public database of the National Center of Biotechnology Information with BioProject ID PRJNA664250. We provided the phenotypic data in Supplementary Table 2 in this version.

Reviewer 2

**>>>General comment**

Wang and his/her many colleagues made efforts to predict heteroisis of rapeseed by pairing parent nucleotide sequences. They developed prediction models by introducing the concept of regional parental genetic-similarity index (PGSI) and successfully reduced dimension in the calculation matrix to give more precise prediction. Moreover, they identified heterosis-QTLs and partitioned the impact of heterosis per subgenome and chromosome. They described a useful approach which is validated through the comparison of field observations and in silico predictions. There are some quite interesting points of this paper which was overall clear and well presented.

**R:** We are thankful for this general comment which is positive and encouraging.

**>>>Main concern**

The authors concluded that the diversity of C subgenome was more important for the rapeseed heterosis than that of A subgenome. I doubted this conclusion. To the best of my knowledge, the A subgenome was much more genetic diverse than the C subgenome. I would, therefore, expect that the A subgenome would be more important for the heterosis than the C genome. The explanation from the authors would be very much appreciated.

**R:** It’s true that the A subgenome was much more diverse than the C subgenome in rapeseed genetic populations, as we previously reported (Wu et al., 2019. Molecular Plant 12:30-43). However, because of the relative conservativeness of the C subgenome, any genetic changes within the C subgenome might result in significant heterosis. Therefore, the C subgenome would be more important than the A subgenome for creating heterosis (Lines 423-432). We don’t think there is a contradiction between the two findings.

I would also list some specifics for your consideration in the revision.

**>>>Minor concern 1**

1. Please mention in the M&M how you calculate high and mid parent heterosis, although I know you defined them in the introduction.

**A1**: We provided the formula to calculate HPH and MPH in the M&M section (Lines 470-475).

**>>>Minor concern 2**

2. Please discuss 10-fold cross-validation. Were there common parents between the training and validation sets?

**A2**: In the 10-fold cross-validation, nine parts of a sample were used to estimate parameters used for the prediction of heterosis in the remaining part of the sample. Eventually, each individual was predicted once and used nine times to estimate the parameters. The 50 female parents of the training set and the validation set are the same, while the male parents are different (Lines 514-518). By now, we’ve performed 10-fold cross-validation with 100 replicates as suggested by Reviewer 3. We discussed the cross-validation a little bit more in the new version (Lines 588-595).

**>>>Minor concern 3**

3. Please list the cross IDs of the top 10% in a table or supplemental table.

**A3:** We listed the cross IDs of the top 10% for each agronomic trait in the Supplementary Table S4 of the new version.

**>>>Minor concern 4**

4. Please include model used in the legend of Fig 5.

**A4:** Fig 5 depicts the predictability of the LASSO_100Kb model. We included the model in the legend of Fig 5 (Lines 851-852).

**>>>Minor concern 5**

5. Fig 6: which correlations was significant?

**A5:** We have added p-values representing the level of significance to Fig 6, and all correlations are significant.

**>>>Minor concern 6**

6. You did not consider LD. You should better discuss the justification and consequences of not considering.

**A6:** LD is a concept of population genetics, meaning the non-random association of alleles at different loci. It is meaningful to calculate LD for a given population of a species. However, it was practically rather difficult to calculate PGSI based on LD, because the linked genes of two parental genomes do not match with each other, and are rather difficult to be determined ( Lines 323-331). Therefore, not considering LD would not result in significant consequences.

Reviewer 3

**>>>General comment**

Reviewer #3: This manuscript describes a interesting study on the heterosis of six agronomic traits in rapeseed. The experiments were carried out using several prediction models and the method of PGSI may reduce the computational load for prediction in the crops with large genomes. In addition, CMS lines and the restorer can be very useful resource for community. Overall, this manuscript provides meaningful results of the heterosis prediction in rapeseed with polyploid genomes.

**R:** Many thanks for recognizing the value of this paper.

There are some comments that the authors may need to address.

**>>>Q1**

Lines 75-77, please add the detail for calculation of the HPH and MPH. For different traits, do you always use the higher values as the better parent for HPH? For some traits, the higher value is not always the expected phenotype, for example, higher plant height may result in logging.

**A1:** As we also responded to the “minor concern 1” from Reviewer 2, we have by now added the details for the calculation of HPH and MPH in the M&M section of the new version (Lines 470-475). Except for the trait of Plant Height (PH), higher values of the other traits such as Thousand-Seed Weight (TSW), Number of Seed per Silique (NSS), Number of Siliques per Plant (NSP), and Number of Branches per Plant (NBP) were better for yield HPH. However, higher PH has not always been better of yield PHP. Higher PH may give rise to higher biomass, which would positively affect yield. On the other hand, e.g. Lodging caused by plant height might negatively influence final yield. In the present study, there was no correlation between PH-HPH and GY-HPH, which could be attributed to relatively low lodging during the seasons when the field experiments were conducted. We discussed this point in the new version (Lines 351-357).

**>>>Q2**

Lines 96-105, the authors mentioned the advantage of GS comparing to MAS, I would expected to discuss somehow in your results. The h-QTLs here are the genomic regions, so it’s really interesting to study how is the situation if only the h-QTLs were used for prediction.

**A2:** (1) We deleted the sentences for the comparison of MAS and GS for a better logic of context. The h-QTL maps published in our study predict the genomic regions (with a dark color) would exert a high impact on the HPH of a trait, suggesting that the PGSI of those regions negatively influences heterosis. We suggested that those genomic regions must be considered in a GS selection for a certain kind of heterosis. We adopted the suggestion to discuss the results in the new version a little bit more (Lines 334-338).

(2) As we described in the method, the variables obtained using the LASSO method were extracted and then re-estimated using linear regression, which is in line with your thought (Lines 567-573).

**>>>Q3**

Lines 153-158, please provide the variance explained by the PC1 and PC2, and please use a different color to show the distribution of 50 CMS lines in Figure S1.

**A3:** By now, we’ve provided the variance explained by PC1 and PC2 in the legend of Figure S1 as well as in lines 866-869. We indicated the distribution of 50 CMS lines in Figure S1 with grey color (Lines 869-871).

**>>>Q4**

Lines 171-175, from the Figure 1, PH in most of hybrids are higher than those in the male parents. As expected, the female parents would affect the hybrids more than the male parents, however the correlations between the hybrids and their male parents were higher. Do you have an explanation or more details about this?

**A4:** According to our experiment, the male parents would affect the hybrids more than the female parents on the height of F1 plants. One of the possible reasons would be the much smaller size of the restorer-line population than that of the CMS-line population. We discussed this possibility in the new version (Lines 357-361).

**>>>Q5**

Lines 179-180, may something be wrong in the figure 2B and table S3 -- NPB or NBP, not consisytent with the manuscript text, please check.

**A5:** In most of the manuscript, we used NBP (for Number of Branches per Plant). In Table S3, however, we also showed the data of NPB (for Number of Primary Branches) and NSP (for Number of Secondary Branches). NBP=NPB+NSP. For uniformity, we now also use NBP in Supplementary Table S5 in the new version.

**>>>Q6**

Lines 198-202, what’s the hypothesis behind this in studying the influence of HPH regardless of the traits and what is the meaning of the results here?

**A6:** The whole paragraph here aimed to explain the correlation between PGSI and F1 heterosis. However, PGSIs were calculated based on different genomic regions, and the heterosis was trait-specific. Here, we intended to mean that the chromosomes with different PGSIs had a different impact (more or less) on trait-specific heterosis. To avoid any misunderstanding, we rephrased the expression in the new version (Lines 199-209).

**>>>Q7**

Line 207, how did you get the h-QTLs? it’s not clear and please provide more details in the method.

**A7:** By now, we’ve made it clearer with details about how we got the *h-*QTLs (Lines 567-573, 612-615).

**>>>Q8**

Lines 208-225, you defined positive and negative effects and both of them will result in higher GY-HPH. What’s the different between positive and negative effects? If the positive effect will result in a higher GY in hybrids?

**A8:** A positive effect indicated with a circle on maps means the smaller the PGSI, the great the heterosis, whereas, a negative effect tagged with a triangle means the bigger the PGSI, the greater the heterosis. The gradation of color (dark or tint) represents the degree of an effect. On the map of *h-*QTLs for GY, the black drop circles indicate a positive effect of the regions on GY-heterosis, which means the smaller the PGSI of the regions, the greater the GY heterosis associated. By now, we have defined positive and negative more clear in the legend of Figure 4 (Lines 846-849).

**>>>Q9**

Lines 227-235, using PGSI to identify the h-QTLs, it decreases the complexity of computational load. As far as I understand, it also decreases the mapping resolution and results in a large genomic region, which as a result increases the number of genes underline the h-QTLs. Then, what’s the advantage to use a high density of marker?

**A9:** We used PGSI to decrease the computational load. However, the higher the SNP density, the more accuracy the PGSI. The LASSO_100Kb model was acceptable for the prediction of heterosis in all six traits (Figure 5). A genomic region with about 100 Kb in size may include 20-25 genes.

**>>>Q10**

Lines 238-242, it’s not clear and please rewrite these sentences.

**A10:** By now, we have deleted this paragraph according to the Question 2 of Reviewer 1.

**>>>Q11**

Line 246, 1860 genes! it’s really a lot. Do you think all of these genes have an important influence on the heterosis of PH?

**A11:** 1860 genes were the sum of genes that were covered by all *h-*QTL regions impacting on plant height. On average, a 100-Kb region of rapeseed genome may consist of 20-25 genes. Therefore, one can expect some 1-2 functional genes underlying an *h-*QTL. The rest genes of the same *h-*QTL likely just link with 1-2 major functional genes, and their allelic changes might not be associated with a certain kind of heterosis. However, it is beyond the scope of this paper to identify the major functional genes of each *h*-QTL. We discussed this point a little bit more in the Discussion section of the new version (Lines 338-344).

**>>>Q12**

Line 277, the cross-validation only with 10 replications, it’s not enough especially for the traits of NSS. What’s the meaning of A,B,C,D… in Figure 5?

**A12:** We have performed cross-validation with 100 replications by now. The results were quite similar as we expected (Figure 5). The A,B,C,D…in Figure 5 indicate statistical significance. Now we indicated this in the new version (Lines 853-854).

**>>>Q13**

Lines 292-294, How did you code the design matrix of additive and dominance effect in your GBLUP_A and GBLUP_AD model? Usually, the dominance effect is related with heterosis, so what’s the explanation of that no difference between the two model?

**A13:** Let $M\text{=\{}M_{jk}\text{\}}$ and $F\text{=\{}F_{jk}\text{\}}$ be $n\times m$ predictor matrices for the male and female parents of the corresponding hybrids, respectively. The additive genotype of the hybrid is defined as, and the dominance genotype is defined as. The corresponding hybrid predictors are defined as

This result is consistent with some studies (Xu et al., 2014. PNAS 11:12456-12461; Cui et al., 2020. Plant Biotechnology Journal 18:57-67), which did not find benefit from adding non-additive variance. This may be because the kinship matrix of additive effect has already captured much information about the kinship matrix of dominant effect. We discussed a little bit more in this respect (Lines 408- 413).

**>>>Q14**

Lines 348-356, it’s a nice discussion about the influence of LD distance. It may be valuable to estimate the LD distance in this population and then to calculate PGSI by considering the LD distance, such as 1(LD distance), 2(LD distance) and 3(LD distance).

**A14:** LD is a concept of population genetics, meaning the non-random association of alleles at different loci. It is meaningful to calculate LD for a given population of a species. However, it was practically rather difficult to calculate PGSI based on LD, because the linked regions of two parental genomes do not match with each other, and are rather difficult to be determined. We discussed this point in the new version (Lines 323- 331).

**>>>Q15**

Line 385, Figure 1 change to Figure S1?

**A15:** Thank you for pointing out this typo (Line 372).

**>>>Q16**

Lines 524-525, it should be in the results part?

**A16:** We removed the sentence to the Results part in the new version (Lines 190-191).

**>>>Q17**

Lines 540-543, were all of the hybrids planted in the 3 years?

**A17:** Yes.

**>>>Q18**

Lines 552-553, why not to adjust the effect of replicates in this model directly instead of using the mean value?

**A18:** We intended to simplify the calculation.

**>>>Q19**

Lines 626-633, it’s not clear that how you got the predictability.

**A19:** We described the method (567-573, 588-595), and provided the codes in getting the predictability as Supplementary data of the manuscript.
